# Spatial distribution and factors associated with adolescent pregnancy in Nigeria: a multi-level analysis

**DOI:** 10.1186/s13690-022-00789-3

**Published:** 2022-01-27

**Authors:** Obasanjo Afolabi Bolarinwa, Zemenu Tadesse Tessema, James Boadu Frimpong, Taiwo Oladapo Babalola, Bright Opoku Ahinkorah, Abdul-Aziz Seidu

**Affiliations:** 1 Department of Public Health Medicine, School of Nursing and Public Health, University of KwaZulu-Natal, Durban, South Africa; 2 Obaxlove consult, Lagos, 100009 Nigeria; 3 Department of Epidemiology and Biostatistics, Institute of Public Health, College of Medicine and Health Sciences, University of Gondar, Gondar, Ethiopia; 4 Department of Health, Physical Education, and Recreation, University of Cape Coast, Cape Coast, PMB TF0494 Ghana; 5 Institute of Governance, Humanities, and Social Sciences, Pan African University, Yaoundé, Cameroon; 6 School of Public Health, University of Technology Sydney, Sydney, NSW 2007 Australia; 7 College of Public Health, Medical and Veterinary Sciences, James Cook University, Townsville, QLD 4811 Australia; 8 Department of Estate Management, Takoradi Technical University, Takoradi, Ghana

**Keywords:** Adolescent pregnancy, Nigeria, DHS, Public health

## Abstract

**Background:**

Adolescent pregnancy is a global public health and social phenomenon. However, the prevalence of adolescent pregnancy varies between and within countries. This study, therefore, sought to investigate the spatial distribution and factors associated with adolescent pregnancy in Nigeria.

**Methods:**

Using data from the women’s recode file, a sample of 9448 adolescents aged 15-19 were considered as the sample size for this study. We employed a multilevel and spatial analyses to ascertain the factors associated with adolescent pregnancy and its spatial clustering.

**Results:**

The spatial distribution of adolescent pregnancy in Nigeria ranges from 0 to 66.67%. A high proportion of adolescent pregnancy was located in the Northern parts of Nigeria. The likelihood of adolescent pregnancy in Nigeria was high among those who had sexual debut between 15 to 19 years [aOR = 1.49; 95%(CI = 1.16-1.92)], those who were currently married [aOR = 67.00; 95%(CI = 41.27-108.76)], and adolescents whose ethnicity were Igbo [aOR = 3.73; 95%(CI = 1.04-13.30)], while adolescents who were currently working [aOR = 0.69; 95%(CI = 0.55-0.88)] were less likely to have adolescent pregnancy.

**Conclusion:**

A high proportion of adolescent pregnancy was located in the Northern parts of Nigeria. In addition, age at sexual debut, educational level, marital status, ethnicity, and working status were associated with adolescent pregnancy. Therefore, it is vital to take cognizant of these factors in designing adolescent pregnancy prevention programs or strengthening existing efforts in Nigeria.

## Background

The health concern and social problem of adolescent pregnancy is not a phenomenon peculiar to a specific region globally; rather, it is prevalent in both high-income and low-and middle-income countries (LMICs), only that the rate varies [1, 2]. In LMICs, for instance, it has been estimated that 21 million girls below 19 years become pregnant every year and 777,000 (6.48%) give birth per year [3]. This trend reflects in the adolescent fertility rate (estimated by the yearly number of births per 1000 adolescent girls) of East Asia, South-East Asia, Central Africa, and West Africa, which are 7.1, 33, 129.5, and 124%, respectively. The estimated rate in Nigeria is currently at 104%, which stands out compared to other African countries [4–6]. However, the increase in adolescent pregnancy rate (currently 106 adolescent births per 1000 population) in Nigeria remains a major concern for the government and other stakeholders [7, 8].

Efforts towards reducing adolescent pregnancy rates have been given attention by the international development communities. For instance, it was key to reaching the millennium development goals (MDGs), which prompted WHO to campaign and advocate for capacity building, productive outcomes, and prevention of early pregnancy among adolescents in LMICs. It has also been central to the global health and the development plans of the current sustainable development goals (SDGs) [2, 9]. Specifically, Goal 3, Target 3.7, seeks to guarantee global access to reproductive health care facilities. Despite these interventions, the problem persists, especially in LMICs, where access to education, information, and healthcare services are limited [10–12].

Furthermore, the implications of adolescent pregnancy have been investigated by different studies. Some of these studies have associated it with social issues such as adolescent labor force involvement rate, inequality, health challenges, low public health disbursement, low number of women in wage employment, and lagging educational achievement [1, 13–16]. However, many authors affirmed that an improved educational system could reduce adolescent pregnancy. It has also been established (empirically) that skills awareness, promotion of contraceptive use, and support for teenage parents are more potent means of curtailing adolescent pregnancy. In addition, the study of Akpor and Thupayagale-Tshweneagae [17] in Nigeria equally noted the potency of pregnancy prevention initiatives at the community level in reducing adolescent pregnancy.

Despite the various interventions and studies on adolescent pregnancy, there has been a paucity of literature on spatial distribution and the associated factors of adolescent pregnancy globally [18, 19] and in Nigeria in particular. Cognizant of this empirical gap, our study has been designed to investigate this. The rationale for choosing spatial distribution tools is to identify locations with a high prevalence of adolescent pregnancy in Nigeria and the associated factors. The multi-level analysis was also employed to identify factors associated with adolescent pregnancy in Nigeria because it has a powerful estimation and is reliable for hierarchical data. The study will stand relevant for different stakeholders and policymakers in Nigeria by identifying locations and regions with the occurrence of the problem at stake. For instance, the findings can be employed to understand the locality where adolescent prevention initiatives should be prioritized in Nigeria. Thus, this study investigated the spatial distribution and factors associated with adolescent pregnancy in Nigeria.

## Methods and materials

### Data source

The 2018 Nigeria Demographic and Health Survey (NDHS) provided the data for this study. The NDHS is a nationally representative survey that collects information on men, women, and children. Data is collected on various topics, including adolescent pregnancy [20, 21]. The NDHS collects data from 36 administrative units and the Federal Capital Territory using a two-stage sampling process. The primary sampling unit for the survey consisted of samples chosen at random from clusters. A total of 9448 teenagers were considered for this investigation, based on data from the women’s recode file. The National Population Commission (NPC) and the International Center for Migration (ICF) have published the detailed methodology of the 2018 NDHS [21]. We followed the guidelines for enhancing the reporting of observational studies in Epidemiology when producing this publication [22]. The dataset can be downloaded from https://dhsprogram.com/data/available-datasets.cfm.

### Outcome variable

The study’s outcome variable was “ever experienced adolescent pregnancy”. The “ever experienced pregnancy” sample consisted of all sexually active females aged 15–19 who were pregnant at the time of the survey or had ever given birth or terminated a pregnancy. Adolescent girls who had ever experienced pregnancy were coded as “yes” and those who had never experienced pregnancy were coded “no”. This coding has been followed in previous studies [23, 24].

### Independent variables

Based on theoretical and practical significance and the availability of the variables in the dataset, we considered both individual and household level factors in our study. The selection of the variables was influenced by their association with adolescent pregnancy in previous studies [23, 24].

### Individual-level factors

The individual-level factors were age of adolescent (years) (15, 16,17,18,19), age at sexual debut (less than 15 years, between 15 and 19), level of education (no education, primary, secondary & above), marital status (never married, currently married, cohabitating, previously married). Working status was generated from the question “currently working”, those who said “yes” were categorised as “working” while those who said “no” were categorized as not working). Other variables were ethnicity (Hausa, Yoruba, Igbo, others), religion (Christianity, Islam, Traditionalist & others), and exposure to media. Exposure to media was categorized as “no” for adolescent girls without exposure to listening to radio, watching television, or reading newspaper, those who had exposure to either of the three variables were categorised as “yes”.

### Household-level factors

The household-level factors were place of residence (urban and rural), wealth index (poorest, poorer, middle, richer, richest), sex of household head (male, female), region (North-Central, North-East, North-West, South-East, South-South, South-West), community literacy level (low, medium, high) and community socioeconomic status (low, medium, high). Community literacy level was generated using the number of people who could read and write based on the clusters. Community socio-economic status was constructed using a household wealth index using the dataset cluster variable. The categorization of these variables into low, medium and high was based on principal component analysis.

### Spatial analysis

To analyse the spatial distribution (geographic variation of adolescent pregnancy), different statistical software like Excel, ArcGIS, and Stata 16 were used. The weighted frequency of outcome variable, the prevalence of adolescent pregnancy (yes/no) with cluster number and geographic coordinate data were merged in Stata 16. Then, the data was exported to an excel CSV delimited format to make the data readable in ArcGIS 10.7 for spatial analysis.

### Spatial autocorrelation analysis

Spatial autocorrelation analysis was done to check whether there is a clustering effect in adolescent pregnancy in Nigeria. This analysis result gives Global Moran’s I value, Z-score, and *P*-value for deciding whether the data is dispersed or random, or clustered. Moran’s I value close to positive 1 indicates a clustering effect, close to negative one indicates dispersed and close to zero random. If *P*-value is significant and I value close to the mean, that adolescent pregnancy had a clustering effect.

### Hot spot analysis (Getis-OrdGi* statistic)

The hot spot analysis tool gives a Getis_Ord or Gi* statistics for a cluster in the dataset. Statistical values like Z-score and *p*-value are computed to determine the statistical significance of clusters. For example, results of the analysis with high GI* value means hot spot areas (high prevalence adolescent pregnancy) and low GI* value means cold spot areas (low prevalence of adolescent pregnancy).

### Spatial interpolation or prediction

Spatial prediction is one of the techniques that estimate unsampled areas based on sampled areas. In Nigeria DHS, a total of 1400 clusters were selected to take a sample for this area that is believed to represent the country. Seven clusters were dropped because they had no longitude and latitude measurements. Thus, a total of 1393 clusters were used for this analysis. Ordinary Kriging prediction methods were used for this study to predict adolescent pregnancy in unobserved areas of Nigeria.

#### Multilevel analysis

Two-level multilevel binary logistic regression models were built to assess the individual and household level variables associated with teen pregnancy in Nigeria. Adolescents were nested within households in the modeling, and subsequently, households were nested within clusters. Clusters were postulated as random effects to account for the unexplained variability at the community level. A total of four models were fitted. Firstly, we fitted an empty model, model 0, which contained no predictors (random intercept). After that, model I contained individual-level variables alone, model II contained household-level variables, and model III was the complete model that contained both individual-level and household-level variables.

The odds ratio and its corresponding 95% confidence intervals were provided for model I-III. These models were fitted by a Stata command “melogit”. The log-likelihood ratio [25], Akaike Information Criteria (AIC) measure, and Schwarz’s Bayesian Information Criteria (BIC) were used for model comparison. The best fit model has the highest log-likelihood and the lowest AIC [26]. We also tested for multicollinearity using variance inflation factor (VIF), which showed no evidence of collinearity among the independent variables. In individual population sample weight (v005/1,000,000) was used in all analyses to account for over-and under-sampling, whereas the “svy” command was used to account for the survey’s complex nature, which also helps in the generalizability of the findings. Stata version 16.0 (Stata Corporation, College Station, TX, USA) was used for statistical analysis.

## Results

### Socio-demographic characteristics of respondents

A total of 8448 adolescents were included in the study. At the individual level, 2078 (24.59%) of the respondents were aged 15. Approximately 6177 (73.12%) of the adolescents had their first sexual intercourse below the age of 15, 5385 (63.74%) had secondary education, and above, 6471 (76.60%) of the respondents were never married, 5388 (63.77%) had mass media exposure. At the household/community level, 4635 (54.86%) of the study adolescents resided in the urban areas, 1810 (21.43%) were from a richer wealth index household, 2737 (32.39%) were residing in North-West, 2973 (35.19%) were from a community with high literacy level, and 5056 (59.85%) were from a community with low socioeconomic status. All the individual and household/community factors showed significant associations with adolescent pregnancy in Nigeria except adolescents working status (Table 1).

**Table 1:** Individual & household-level characteristics of respondents by adolescent pregnancy in Nigeria

Variable	Weighted frequency	Weighted percentage	Adolescent pregnancy (%)		*p*-value (χ^2^)
Individual-level variables			No	Yes	
**Adolescent current age**					*p* < 0.001
15	2078	24.59	98.32	1.68	
16	1585	18.76	95.81	4.19	
17	1579	18.69	93.45	6.54	
18	1920	22.73	89.97	10.03	
19	1286	15.22	89.43	10.57	
**Age at sexual debut**					*p* < 0.001
Less than 15 years	6177	73.12	97.86	2.14	
Between 15 and 19	2271	26.88	82.34	17.66	
**level of education**					*p* < 0.001
No Education	2182	25.83	84.85	15.15	
Primary	880	10.42	92.75	7.25	
Secondary & above	5385	63.74	97.43	2.57	
**Marital status**					*p* < 0.001
Never married	6471	76.60	99.53	0.47	
Currently Married	1870	22.14	74.08	25.92	
Cohabitating	56	0.67	70.41	29.59	
Previously Married	50	0.60	97.7	2.30	
**Working status**					0.14
Not working	5441	64.41	93.36	6.64	
Working	3007	35.59	94.29	5.71	
**Ethnicity**					
Hausa	3368	39.86	88.96	11.04	
Yoruba	1118	13.24	98.36	1.64	
Igbo	1176	13.92	97.69	2.23	
Others	2786	32.98	95.84	4.16	
**Religion**					*p* < 0.001
Christianity	3528	41.76	97.59	2.41	
Islam	4879	57.75	90.87	9.13	
Traditionalist & others	42	0.49	93.29	6.71	
**Exposure to media**					*p* < 0.001
No	3060	36.23	89.84	10.16	
Yes	5388	63.77	95.88	4.12	
**Household-level**					
**Place of residence**					*p* < 0.001
Urban	3813	45.14	97.31	2.69	
Rural	4635	54.86	90.71	9.29	
**Wealth index**					*p* < 0.001
Poorest	1427	16.89	89.36	10.64	
Poorer	1740	20.59	88.28	11.72	
Middle	1758	20.81	94	6.00	
Richer	1810	21.43	97.01	2.99	
Richest	1713	20.28	98.96	1.04	
**Sex of household head**					*p* < 0.001
Male	6929	82.02	92.84	7.16	
Female	1519	17.98	97.58	2.42	
**Region**					*p* < 0.001
North Central	1183	14.00	94.85	5.15	
North East	1497	17.73	92.70	7.30	
North West	2737	32.39	89.05	10.95	
South East	928	10.98	97.74	2.26	
South South	888	10.51	97.61	2.39	
South West	1215	14.38	98.27	1.73	
**Community literacy level**					*p* < 0.001
Low	2880	34.09	86.66	13.34	
Medium	2595	30.72	95.69	4.31	
High	2973	35.19	93.69	1.25	
**Community socioeconomic status**					*p* < 0.001
Low	5056	59.85	90.61	9.39	
Medium	310	3.67	96.84	3.16	
High	3082	36.48	98.42	1.58	

NDHS, 2018

### Spatial analysis

#### Spatial distribution of adolescent pregnancy

**Fig. 1:**
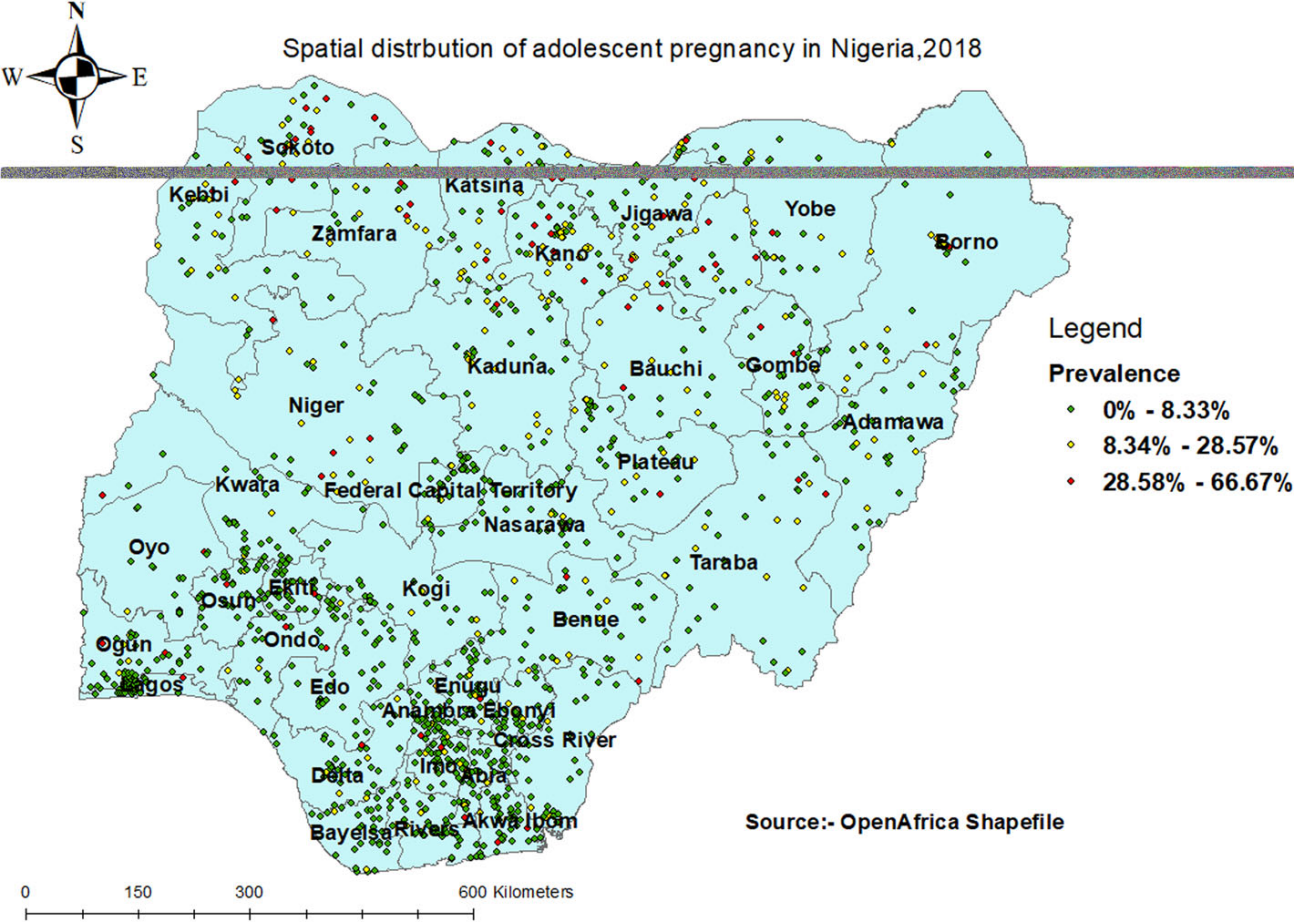
Spatial distribution of adolescent pregnancy in Nigeria, 2018

The spatial distribution of adolescent pregnancy in Nigeria ranges from 0 to 66.67%. A high proportion of adolescent pregnancy was located in Northern parts of Nigeria (Fig. 1).

#### Spatial autocorrelation

**Fig. 2:**
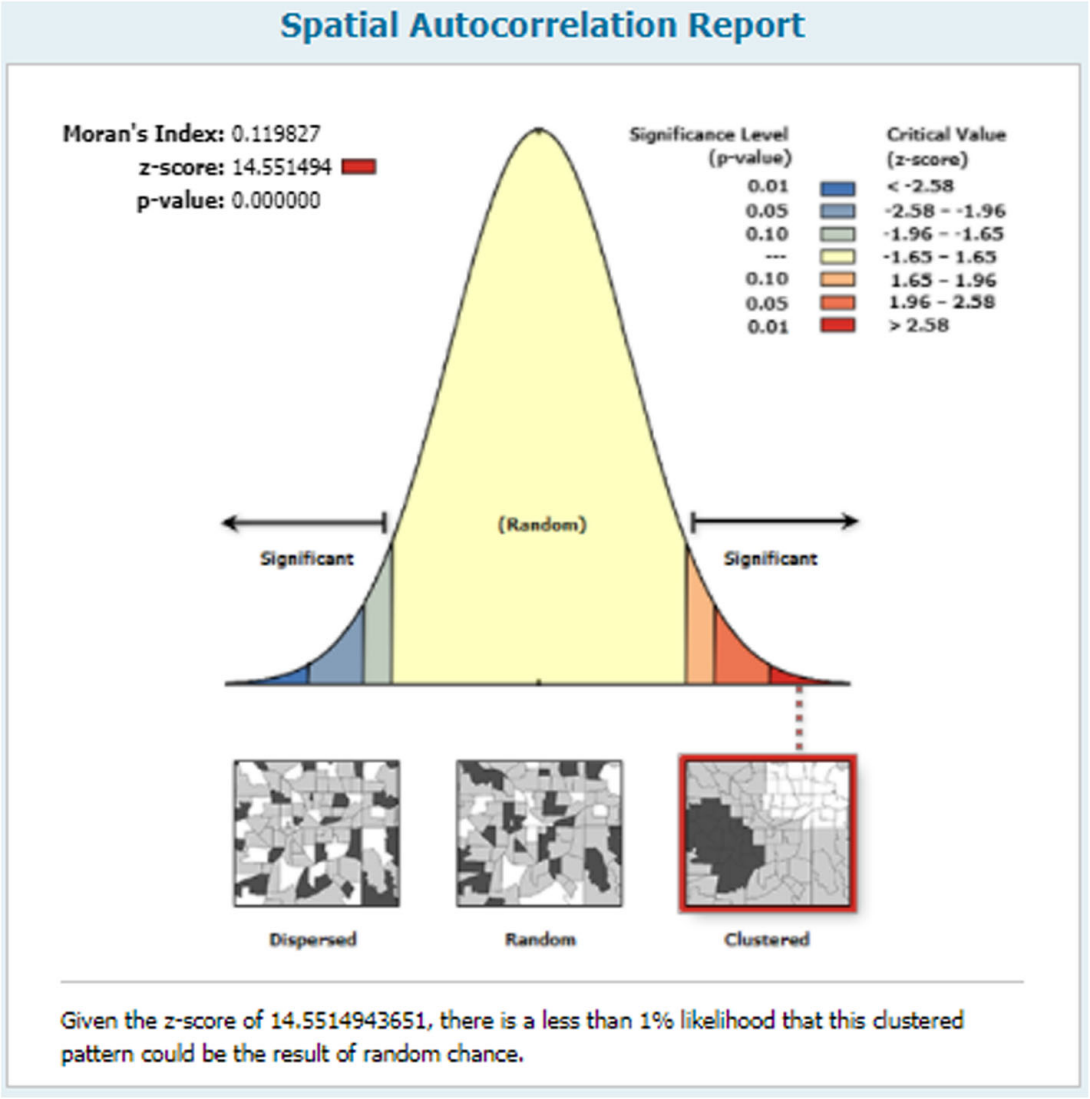
Spatial autocorrelation of adolescent pregnancy in Nigeria, 2018

The spatial autocorrelation of adolescent pregnancy was clustered in Nigeria across all regions. The spatial autocorrelation analysis result revealed that Moran’s I value 0.119, Z-score 14.55 and *p*-value < 0.001 indicated that adolescent pregnancy in Nigeria was clustered (Fig. 2).

### Hot spot analysis

**Fig. 3:**
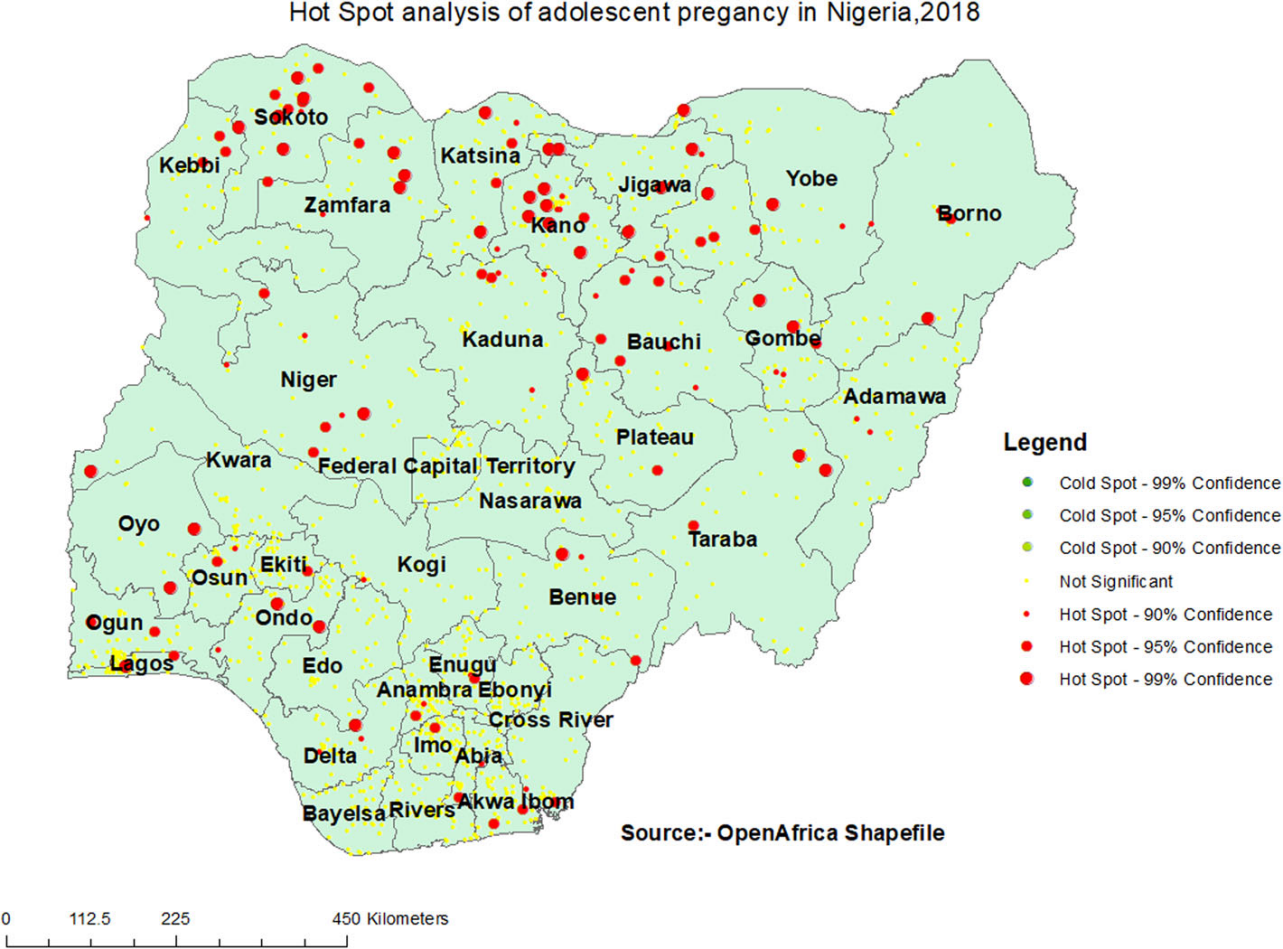
Hot spot analysis of adolescent pregnancy in Nigeria, 2018

Hot spot analysis was done using Getis ord GI* analysis to detect hot and cold spot areas. Hot spot areas (high proportion of adolescent pregnancy) were located in Sokoto, Kebbi, Zamfara, Katsina, Kano, Jigawa, Bauchi and Niger (Fig. 3).

### Predictors of adolescent pregnancy

#### Spatial interpolation

**Fig. 4:**
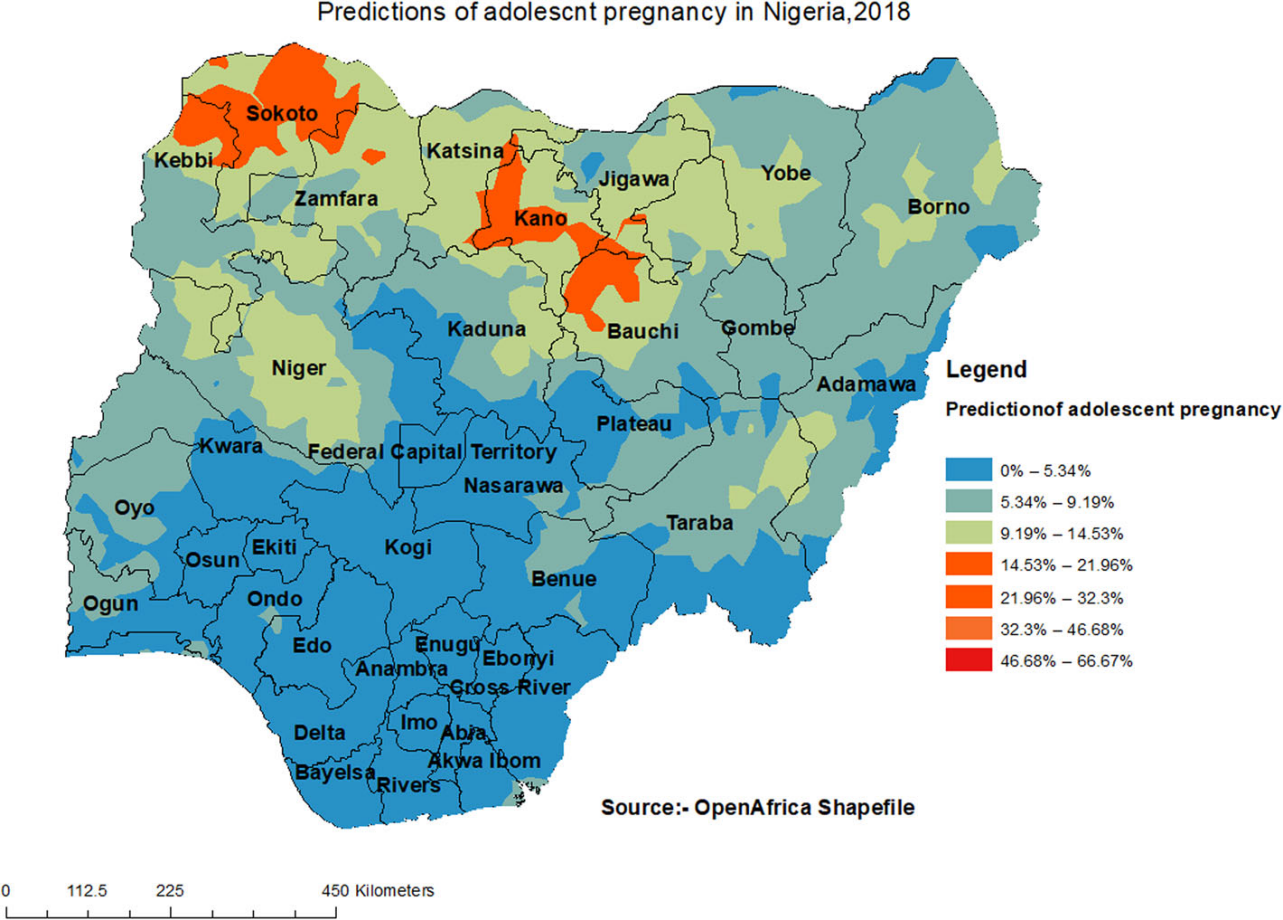
Interpolation of adolescent pregnancy in Nigeria, 2018

The interpolation analysis was done using Ordinary kriging interpolation. This analysis gives predictions for unsampled areas based on sampled areas. The interpolation result revealed that Kebbi, Sokoto, Kano, Bauchi, and Katsina had a higher proportion of adolescent pregnancy (Fig. 4).

#### Multi-level fixed effects (measures of associations) results

The factors associated with adolescent pregnancy in Nigeria include age at sexual debut, educational level, marital status, ethnicity, and working status.

At the individual-level factors, the likelihood of adolescent pregnancy in Nigeria was high among those who had sexual debut between 15 to 19 years [aOR = 1.49; 95%(CI = 1.16-1.92)], and adolescents who were currently married [aOR = 67.00; 95%(CI = (41.27-108.76)], and adolescents whose ethnicity was Igbo [aOR = 3.73; 95%(CI = 1.04-13.30)], while adolescents who were currently working [aOR = 0.69; 95%(CI = 0.55-0.88)], were less likely to have adolescent pregnancy.

#### Random effects (measures of variations) results

The empty model (Model 0), as shown below in Table 2, depicted a substantial variation in the likelihood of adolescent pregnancy in Nigeria across the Primary Sampling Units (PSUs) clustering [σ2 = 1.09; 95% (CI = 0.79–1.51)]. Model 0 indicated that 25% of the variation in adolescent pregnancy in Nigeria was attributed to the variation between Intra-Class Correlation [27], i.e., (ICC = 0.25). The variation between-cluster decreased to 1% (0.01) in Model I (individual level only). In the household/community-level only (Model II), the ICC increased to 8%, while the ICC of the complete model with both the individual and household/community factors (Model III) declined to 1%. This further reiterates that the variations in the likelihood of adolescent pregnancy in Nigeria are attributed to the clustering variation in PSUs. The Akaike’s Information Criterion (AIC) and Schwarz’s Bayesian Information Criteria (BIC) values showed a successive reduction, which means a substantial improvement in each of the models over the previous model and also affirmed the goodness of Model III developed in the analysis. Therefore, Model III, the complete model with both the selected individual and household/community factors, was selected to predict the likelihood of adolescent pregnancy in Nigeria.

**Table 2:** Multilevel logistic regression models for individual and household/community predictors of adolescent pregnancy in Nigeria

Variables	Model 0	Model I	Model II	Model III
		aOR [95% CI]	aOR [95% CI]	aOR [95% CI]
**Fixed effects results**				
Individual-level variables				
**Adolescent current age**				
15		RC		RC
16		1.08 [0.66-1.77]		1.09 [0.67-1.78]
17		0.94 [0.59-1.48]		0.95 [0.60-1.50]
18		1.24 [0.81-1.92]		1.25 [0.81-1.94]
19		1.06 [0.68-1.67]		1.10 [0.70-1.74]
**Age at sexual debut**				
Less than 15 years		RC		RC
Between 15 and 19		1.50** [1.16-1.92]		1.49** [1.161.92]
**level of education**				
No Education		RC		RC
Primary		1.27 [0.91-1.76]		1.28 [0.92-1.80]
Secondary & above		1.27 [0.93-1.75]		1.43* [1.00-2.03]
**Marital status**				
Never married		RC		RC
Currently Married		70.71*** [44.12-113.32]		67.00*** [41.27-108.76]
Cohabitating		79.48*** [40.04-157.74]		69.35*** [34.16-140.80]
Previously Married		7.85** [1.77-34.71]		7.91** [1.78-35.19]
**Working status**				
Not working		RC		RC
Working		0.72** [0.57-0.91]		0.69** [0.55-0.88]
**Ethnicity**				
Hausa		RC		RC
Yoruba		1.72 [0.92-3.22]		1.38 [0.53-3.57]
Igbo		1.68 [0.92-3.04]		3.73* [1.04-13.30]
Others		1.18 [0.88-1.58]		1.31 [0.93-1.84]
**Religion**				
Christianity		RC		RC
Islam		1.29 [0.87-1.91]		1.37 [0.89-2.10]
Traditionalist & others		2.17 [0.50-9.40]		2.00 [0.45-8.80]
**Exposure to media**				
No		RC		RC
Yes		0.83 [0.66-1.05]		0.86 [0.67-1.09]
Household-level				
**Place of residence**				
Urban			RC	RC
Rural			1.10 [0.82-1.48]	0.87 [0.63-1.20]
**Wealth index**				
Poorest			RC	RC
Poorer			1.36* [1.06-1.75]	1.08 [0.83-1.41]
Middle			1.09 [0.80-1.49]	1.05 [0.75-1.47]
Richer			0.94 [0.63-1.42]	0.87 [0.55-1.37]
Richest			0.37** [0.18-0.77]	0.47 [0.21-1.04]
**Sex of household head**				
Male			RC	RC
Female			0.48*** [0.33-0.70]	0.89 [0.58-1.35]
**Region**				
North Central			RC	RC
North East			1.47* [1.05-2.07]	1.93 [0.64-1.37]
North West			2.18*** [1.59-2.98]	1.11 [0.74-1.66]
South East			1.03 [0.60-1.75]	0.52 [0.14-1.96]
South South			1.26 [0.73-2.17]	1.15 [0.60-2.20]
South West			1.43 [0.83-2.45]	1.73 [0.71-4.21]
**Community literacy level**				
Low			RC	RC
Medium			0.46*** [0.35-0.61]	0.94 [0.68-1.30]
High			0.24*** [0.16-0.38]	0.88 [0.52-1.48]
**Community socioeconomic status**				
Low			RC	RC
Medium			0.43 [0.16-1.12]	0.56 [0.68-1.30]
High			0.85 [0.55-1.32]	0.90 [0.55-1.48]
**Random effects results**				
PSU Variance (95% CI)	1.09 [0.79-1.51]	0.03 [0.00-10.36]	0.27 [0.12-0.60]	0.03 [0.00-16.42]
ICC	0.25	0.01	0.08	0.01
LR Test	χ2 = 84.61, *p* < 0.001	χ2 = 0.12, *p* > 0.05	χ2 = 8.53, *p* < 0.01	χ2 = 0.10, p > 0.05
Wald χ2	**Reference**	525.05***	261.32***	523.29***
**Model fitness**				
Log-likelihood	− 1840.77	− 1207.84	− 1680.80	− 1199.78
AIC	3685.54	2453.68	3395.60	2467.56
BIC	3699.61	2587.42	3515.26	2706.88
Number of clusters	1363	1363	1363	1363

Weighted NDHS, 2018

Model 0 is the null model, a baseline model without any explanatory variable

Model, I is adjusted for individual-level variables (Adolescent age, age at sexual debut, educational level, marital status, working status, ethnicity, religion, and media exposure)

Model II is adjusted for household/community level variables (Place of residence, wealth index, region, sex of household head, community literacy level, and community socioeconomic status)

Model III is the final model adjusted for both individual and household/community level variables

*Exponentiated coefficients* 95% confidence intervals in brackets, *AOR* adjusted Odds Ratios, *CI* Confidence Interval, *RC* Reference Category, *PSU* Primary Sampling Unit, *ICC* Intra-Class Correlation, *LR Test* Likelihood ratio Test, *AIC* Akaike’s Information Criterion, *BIC* Schwarz’s Bayesian Information Criteria

*p < 0.05; **p < 0.01; ***p < 0.001

## Discussion

This study investigated the spatial distribution and factors associated with adolescent pregnancy in Nigeria using the 2018 NDHS. The study found that the spatial distribution of adolescent pregnancy in Nigeria ranged from 0 to 66.67%. A high proportion of adolescent pregnancies were located in Sokoto, Kebbi, Zamfara, Katsina, Kano, Jigawa, Bauchi, and Niger. A possible reason for this finding could be the high level of poverty, predisposing adolescents to engage in premarital sexual relations to meet their needs [28]. It could also be that a low level of education among adolescents in the Northern part of Nigeria predisposes them to indiscriminate sexual relations with older men, resulting in unwanted pregnancies [29]. Another possible reason for this finding could be early marriage (less than 18 years) among adolescents, which reduces their autonomy to negotiate for safer sex, increasing their likelihood of getting pregnant in their adolescence stage [30]. Maigari [31] examined the changing rates of early marriages in northern Nigeria and found that these changing dynamics of early marriages were as a result of increased rates of poverty in Nigeria, with over 39% of Nigeria living below the international poverty line of $1.90 per day [32].

Similar to the finding of a previous study [33], the study found that the likelihood of adolescent pregnancy in Nigeria was high among those who had sexual debut between 15 to 20 years. A plausible reason for this finding could be that adolescents at that age face the challenge of affording their basic needs and contraceptives, exposing them to having sexual relationships with older men to cater for such needs [34]. It is also possible that their peers influenced adolescents who had their sexual debut between 15 to 20 years to engage in sexual relationships with elderly men to cater for their needs [34]. Another possible reason could be the lack of parental counseling and guidance, increasing the likelihood of female adolescents being involved in premarital sexual intercourse [34].

Access to family planning services among adolescents is limited, and this is due to several factors, including individual factors (such as fear of side effects, poor knowledge and lack of awareness, low self-esteem, etc.), health systems (such as lack of privacy, judgmental attitude of health workers, inadequate supply of contraceptive), interpersonal factors (such as the negative attitude of parents towards sexuality education, poor communication on matters of sexual and reproductive health) [35, 36]. A study concluded that the prevalence of modern contraceptive use among Nigerian adolescents is 7.8% [37]. Also, cultural norms and religious practices bar adolescents from having premarital sex and also accessing family planning services [35]. A study revealed that the median age for sexual debut for females and males was 16 years and 17 years, respectively [38].

It is a fact that adolescents who have higher educational levels are protected from unwanted pregnancies due to the empowerment that higher education comes with [39, 40]. However, it is surprising that this study found that the likelihood of adolescent pregnancy in Nigeria was high among adolescents who had secondary education and above. This finding contradicts that of a previous study [41]. An acceptable explanation for this finding could be that adolescents who have attained higher education have reduced their utilization of contraceptives, increasing their likelihood of getting pregnant [42, 43]. Another reason for this finding could be that programs that help reduce adolescent pregnancy have neglected adolescents who have attained higher education, leading to their increased likelihood of getting pregnant.

Adolescents who were currently married were more likely to have adolescent pregnancy. The study’s finding is similar to previous studies [1, 23, 24]. A possible reason for this finding is that married adolescents are more likely to be predisposed to getting pregnant, resulting from the increase in the desire to have children [24]. Another possible reason could be the pressure given to female adolescents to marry early and a subsequent expectation of getting pregnant [44]. For instance, a qualitative study conducted in northern Nigeria by Maigari [31] revealed that the parents of teenagers believed that when teenage girls are left unmarried, they may bring shame to the family after getting pregnant out of wedlock which is seen as a taboo; hence they are pushed into marriage early. As a way of addressing the problem of early marriage in Nigeria, the Federal Government in 2003 passed the Child Rights Act that criminalizes marriage below age 18, yet some states are yet to institute the policy as at 2020 [45].

The likelihood of adolescent pregnancy in Nigeria was high among adolescents whose ethnicity was Igbo. A possible reason for this finding could be that adolescents who are with the Igbo ethnic group have multiple sexual partners, increasing their likelihood of getting pregnant [38]. Another possible reason for this finding could be the low access and utilization of contraceptives among adolescents with the Igbo ethnic group, increasing their likelihood of getting pregnant [46, 47]. Oluwasanu [35] specifically found that only 8.7, 7.8 and 5.8% of women used condoms, intrauterine devices (IUDs) and pills, respectively. The patriarchal tradition of the Igbo ethnic group where men are perceived as superior and more domineering than women could also be possible for this finding, as the female adolescent who is in a sexual relationship with the men is least expected to decline sexual intercourse with them [48]. More qualitative studies are warranted to further understand this particular finding.

Corroborating the finding of a previous study [41], this study found that adolescents who were currently working were less likely to have adolescent pregnancy. A possible reason for this finding could be that those working are economically independent of catering for their needs, reducing their likelihood of engaging in sexual relations with older men for financial support [41]. Moreover, it could be that adolescents who are working are more empowered to negotiate for safer sex, reducing their likelihood of getting pregnant [41].

### Implications for policy and public health practice

Findings on the effect of early sexual debut on adolescent pregnancy imply the need to offer sexual and reproductive health education on the negative effects of early sexual debut. To reduce the effect of child marriage on adolescent pregnancy, there is a need for community sensitization and education towards eliminating child marriage in Nigeria. The finding on the working status and adolescent pregnancy implies that providing adolescents with employment could significantly reduce the rates of adolescent pregnancy in Nigeria, hence, governmental and non-governmental organizations who are involved in the fight against adolescent pregnancy should consider providing vocational training for female adolescents. The government and non-governmental organisations should also consider implementing the existing guidelines in line with what is recommended in this study because this will help reduce adolescent pregnancy in Nigeria.

### Strengths and limitations

Our study has several strengths. First, nationally representative data supports the generalizability of the findings to women in Nigeria. Additionally, the use of geographical information system (GIS) in the analysis of the spatial distribution enabled us to identify the hotspots of adolescent pregnancy in Nigeria, and this is a major contribution to the literature on adolescent pregnancy in Nigeria. Moreover, identifying these adolescent pregnancy hotspots would benefit program designers and implementers in their design on context-specific and population-targeted interventions to reduce adolescent pregnancy.

Nevertheless, the study was not without some limitations. A major limitation to this study was that the data used was cross-sectional in design, limiting us from establishing causality. Also, the data was self-reported, making it highly susceptible to recall bias and social desirability bias. Another limitation is that the survey data used was conducted in the year 2018, and the outcome of this study might not necessarily represent the current situation in Nigeria. The wide, confident interval in marital status reported in this study is because of the skewness of the variable due to the large number of the adolescent are unmarried. The wide, confident interval in marital status reported in this study is because of the skewness of the variable due to a large number of the adolescent being unmarried.

## Conclusion

The study investigated the spatial distribution and factors associated with adolescent pregnancy in Nigeria using the 2018 NDHS. We found that the spatial distribution of adolescent pregnancy in Nigeria ranged from 0 to 66.67%. A high proportion of adolescent pregnancies were located in Sokoto, Kebbi, Zamfara, Katsina, Kano, Jigawa, Bauchi, and Niger. Age at sexual debut, educational level, marital status, ethnicity, and working status were the factors associated with adolescent pregnancy. Therefore, it is vital to take cognizant of these factors in designing adolescent pregnancy prevention programs or strengthening existing efforts in Nigeria.

## Data Availability

The datasets utilized in this study can be accessed at https://dhsprogram.com/data/available-datasets.cfm.

## Abbreviations

WHO: World Health Organization
UNFPA: United Nations Population Fund
UNDESA: United Nations Department of Economic and Social Affairs
MDGs: Millennium development goals
SDGs: Sustainable development goals
NDHS: Nigeria Demographic and Health Survey
NPC: National Population Commission
ICF: International Center for Migration
LLR: Log-likelihood ratio
AIC: Akaike Information Criteria
BIC: Schwarz’s Bayesian Information Criteria
VIF: Variance inflation factor
ICC: Intra-class correlation
GIS: Geographical information system
